# Supplementary material for: Cytoplasmic localization of PUS7 facilitates a pseudouridine-dependent enhancement of cellular stress tolerance
Source: bioRxiv. 2025 Sep 22:2025.09.22.675887. Preprint. [Version 1] doi: 10.1101/2025.09.22.675887 (PMC12485686; doi:10.1101/2025.09.22.675887)
Supplement: Supplement 1 [file media-1.pdf]

Cytoplasmic localization of PUS7 facilitates a pseudouridine-dependent enhancement of cellular stress tolerance

Minli Ruan[1], Sean M. Engels[2], Matthew R. Burroughs[2], Xiaoyan Li[3], Rosella Stower[1], Talia Tzadikario[4], Connor Powell[4], Dylan Bloch[4], Oleksandra Fanari[4], Stuart Akeson[4], Daniel E. Eyler[3], Chase A. Weidmann[1], Sara Rouhanifard[4], Miten Jain[4], Lydia M. Contreras[2], Kristin S. Koutmou[1,3]*

[1]University of Michigan, Department of Biological Chemistry, Ann Arbor, MI 48109

[2]University of Texas, McKetta Department of Chemical Engineering, Austin, TX 78712

[3]University of Michigan, Department of Chemistry, Ann Arbor, MI 48109

[4]Northeastern University, Department of Bioengineering, Boston, MA 02120

*kkoutmou@umich.edu, 930 N University, Ann Arbor, MI 48109, (734) 764-5650

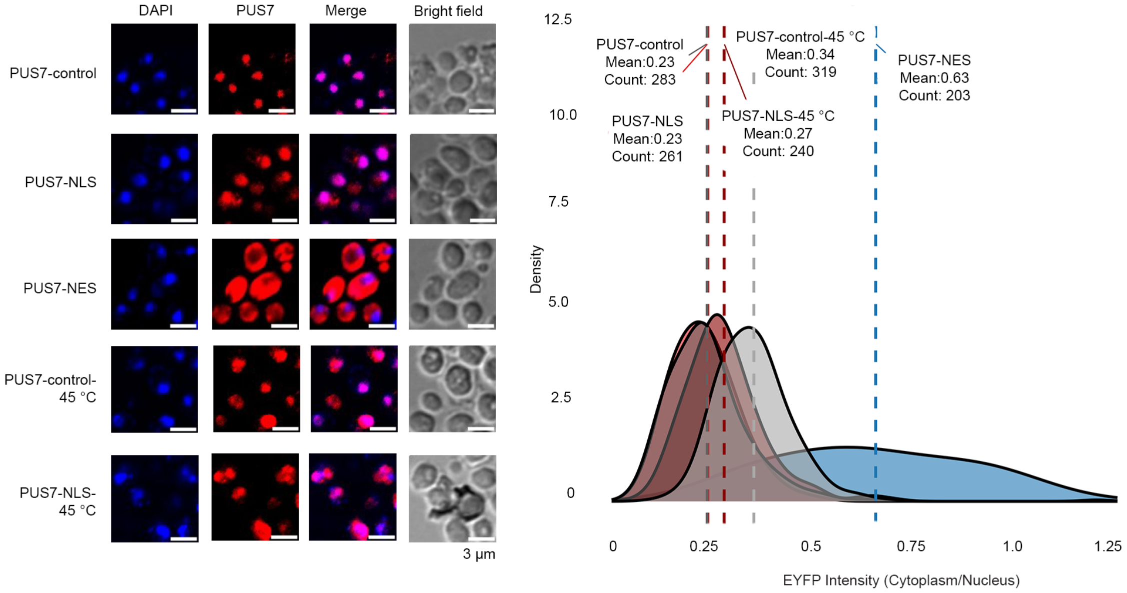

Supplementary Fig. 1 | Immunofluorescence of anti-FLAG showing localization of PUS7-control, -NLS, and -NES fused PUS7 as well as PUS7-control and -NLS under heat shock (heat shock (45 °C for 15 min)), together with quantification of the Fluorescence Intensity (FI) ratio in the cytoplasm *vs* in the nucleus (n>100 cells). Scale bar: 3 μm.

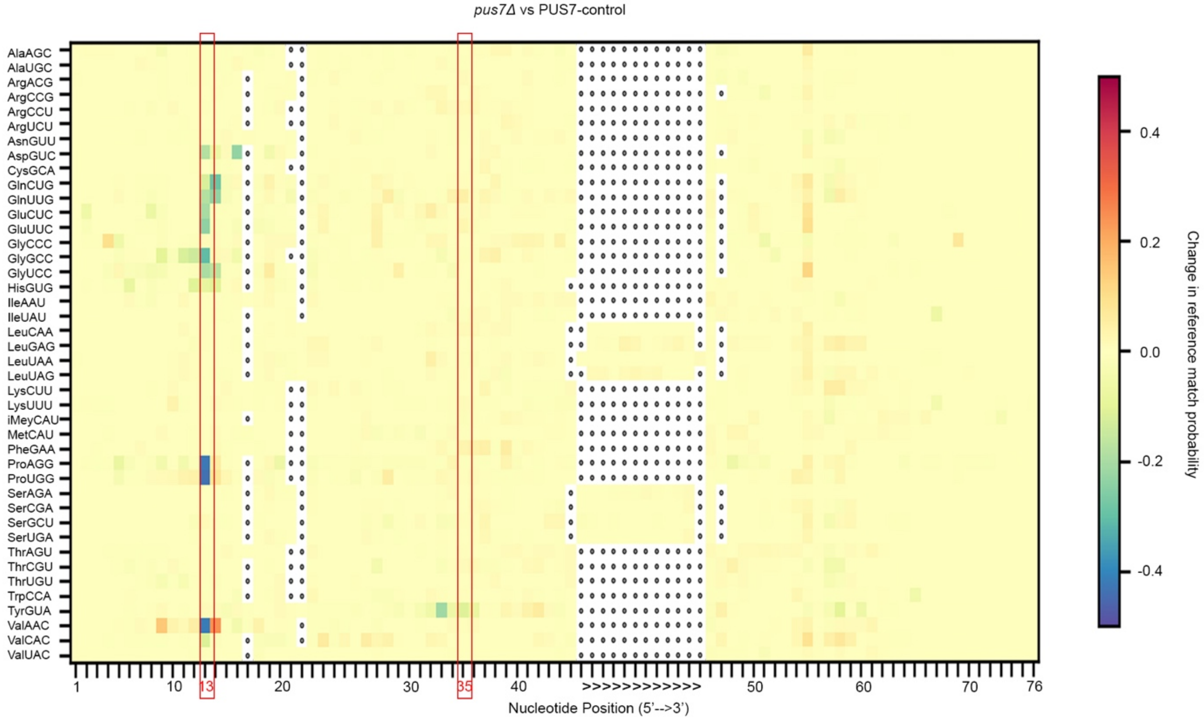

Supplementary Fig. 2 | Ψ13 and Ψ35 modifications appear upregulated in PUS7-control compared to *pus7*Δ cells, highlighted in red boxes.

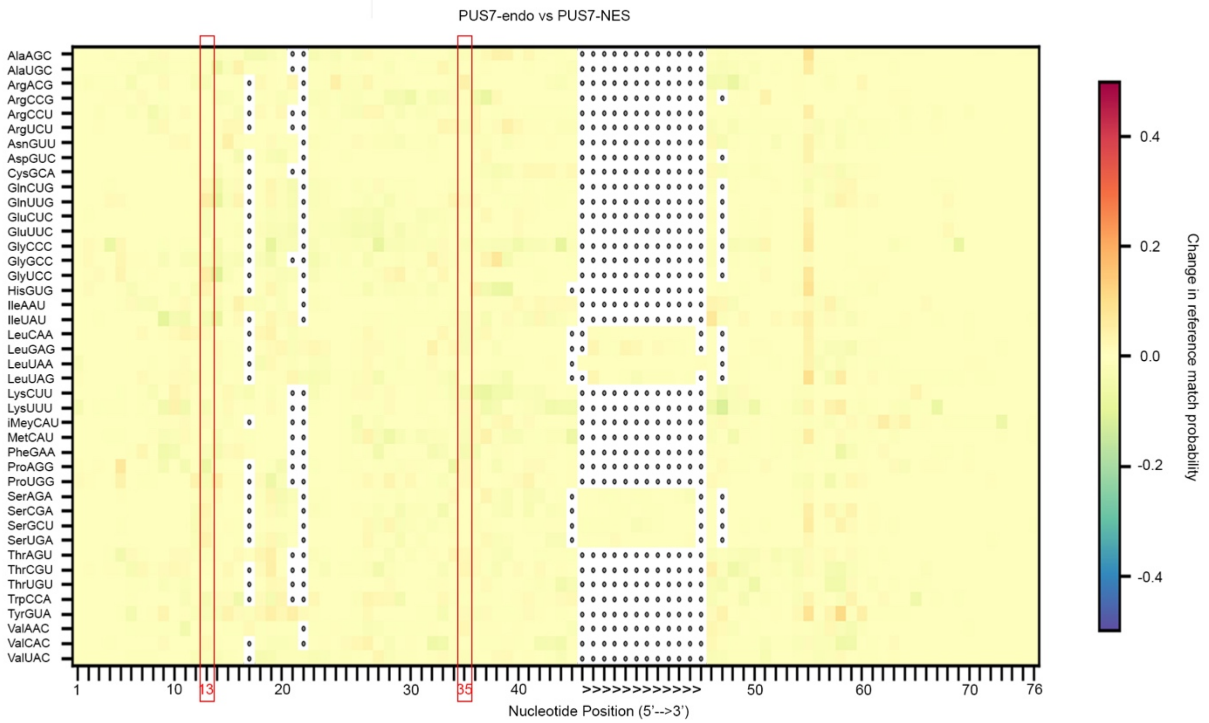

Supplementary Fig. 3 | Ψ incorporation on tRNA positions targeted by PUS7 in eukaryotes (Ψ13 and Ψ35) is unaltered between cells expressing only endogenous PUS7 and PUS7-NES, highlighted in red boxes.

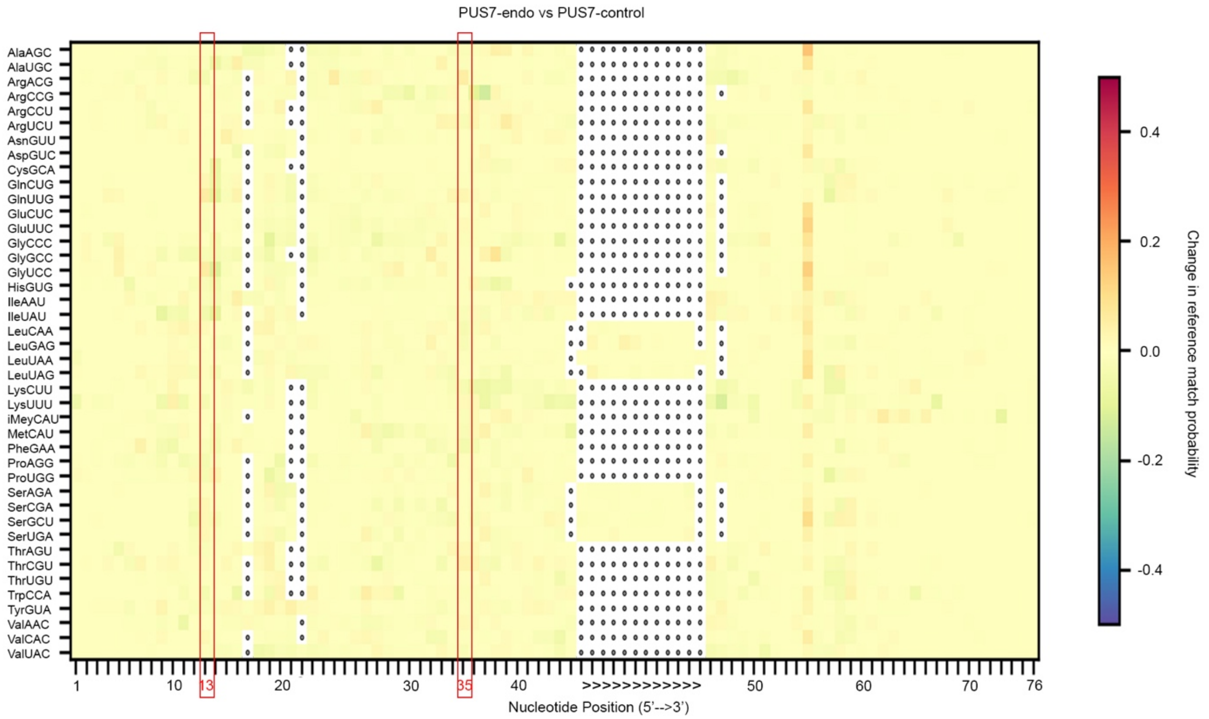

Supplementary Fig. 4 | Ψ incorporation on tRNA positions targeted by PUS7 in eukaryotes (Ψ13 and Ψ35) is unaltered between cells expressing only endogenous PUS7 and PUS7-control, highlighted in red.

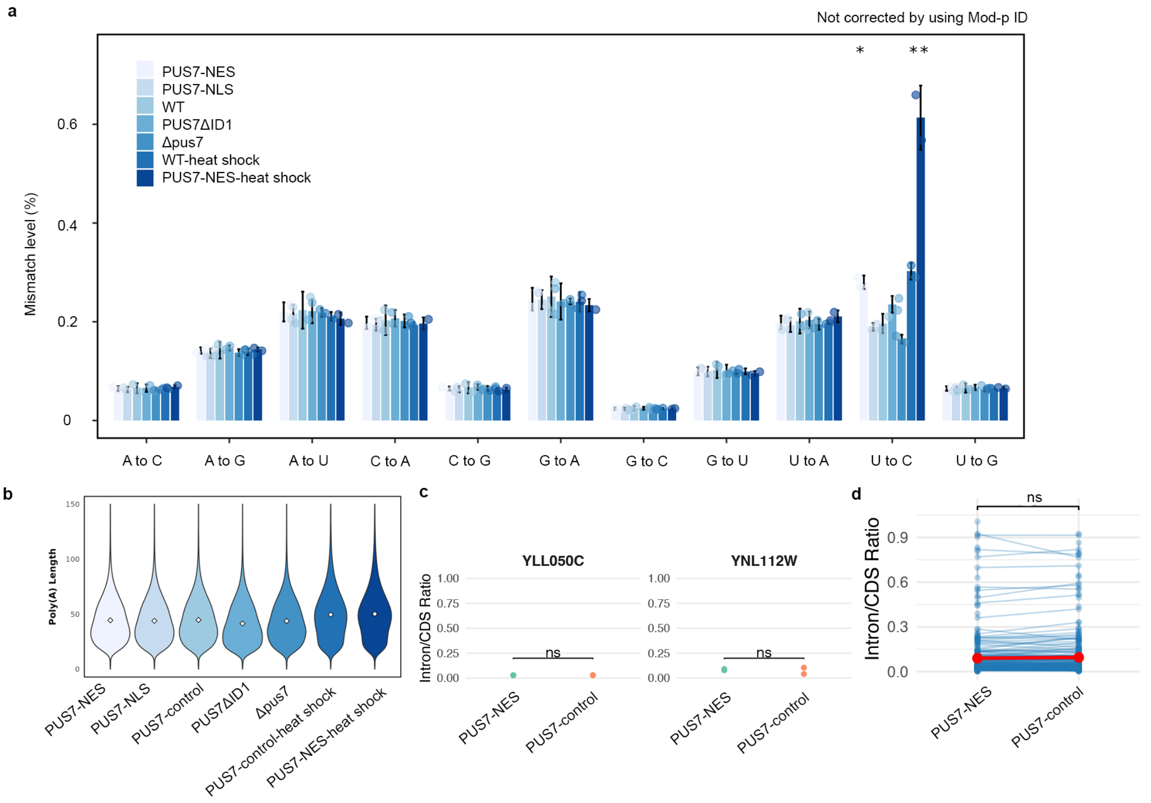

Supplementary Fig. 5 | U-to-C mismatch level changes observed in different cell groups without affecting poly(A) length or pre-mRNA splicing. a, Nucleotide mismatch levels for all four nucleotides across different cell groups from RNA002. Student's two-sample t-test (unpaired, two-tailed) with Bonferroni correction was used to calculate adjusted p-values. Significant U-to-C mismatch increases were observed in PUS7-NES ($p = 0.038$), PUS7-FLAG-heat shock ($p = 0.029$), and PUS7-NES-heat shock ($p = 0.013$). Data are shown as mean ± SD. (*$P < 0.05$; $n = 2$). **b**, polyA length is not getting affected among different groups. **c**, PUS7 relocalization does not broadly affect RNA splicing. Intron/CDS read ratios are shown for COF1/YLL050C (Ψ site detected in PUS7-control) and DBP2/YNL112W (Ψ site detected in PUS7-NES); paired, two-tailed t-test, $n = 2$. **d**, Overall splicing levels of 172 genes containing introns are similar between PUS7-NES and PUS7-control ($n = 172$).

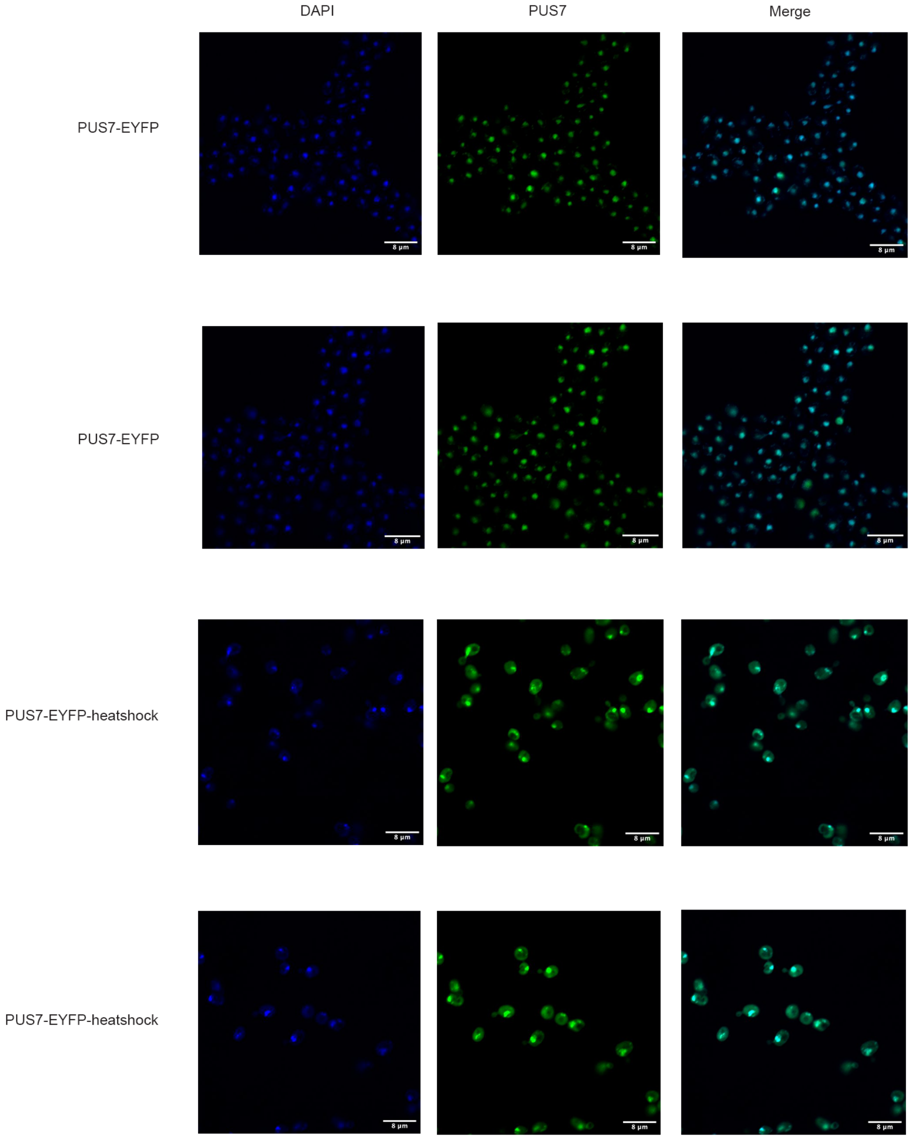

Supplementary Fig. 6 | More images showing PUS7-EYFP relocalization under no stress conditions and heat shock (45 °C, 1h). Scale bar: 8 µm.

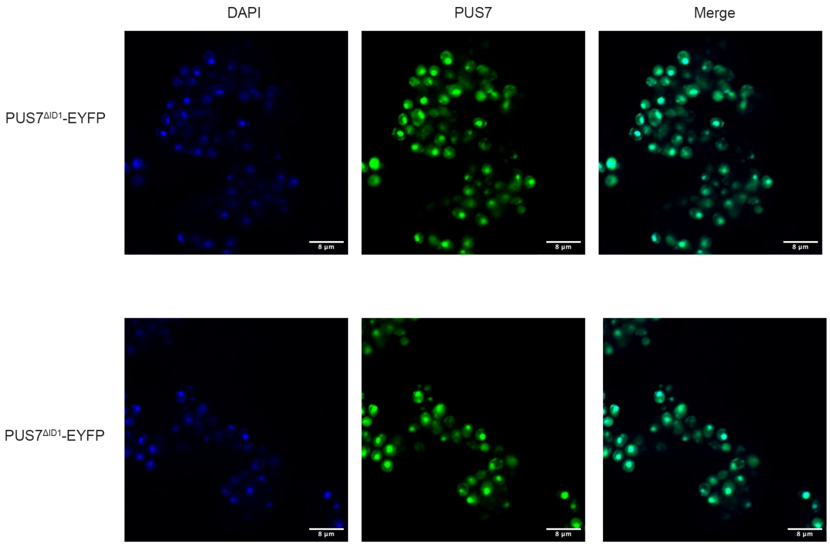

Supplementary Fig. 7 | More images showing PUS7 relocalization for cell expression PUS7$^{\Delta ID1}$-EYFP. Scale bar: 8 μm.

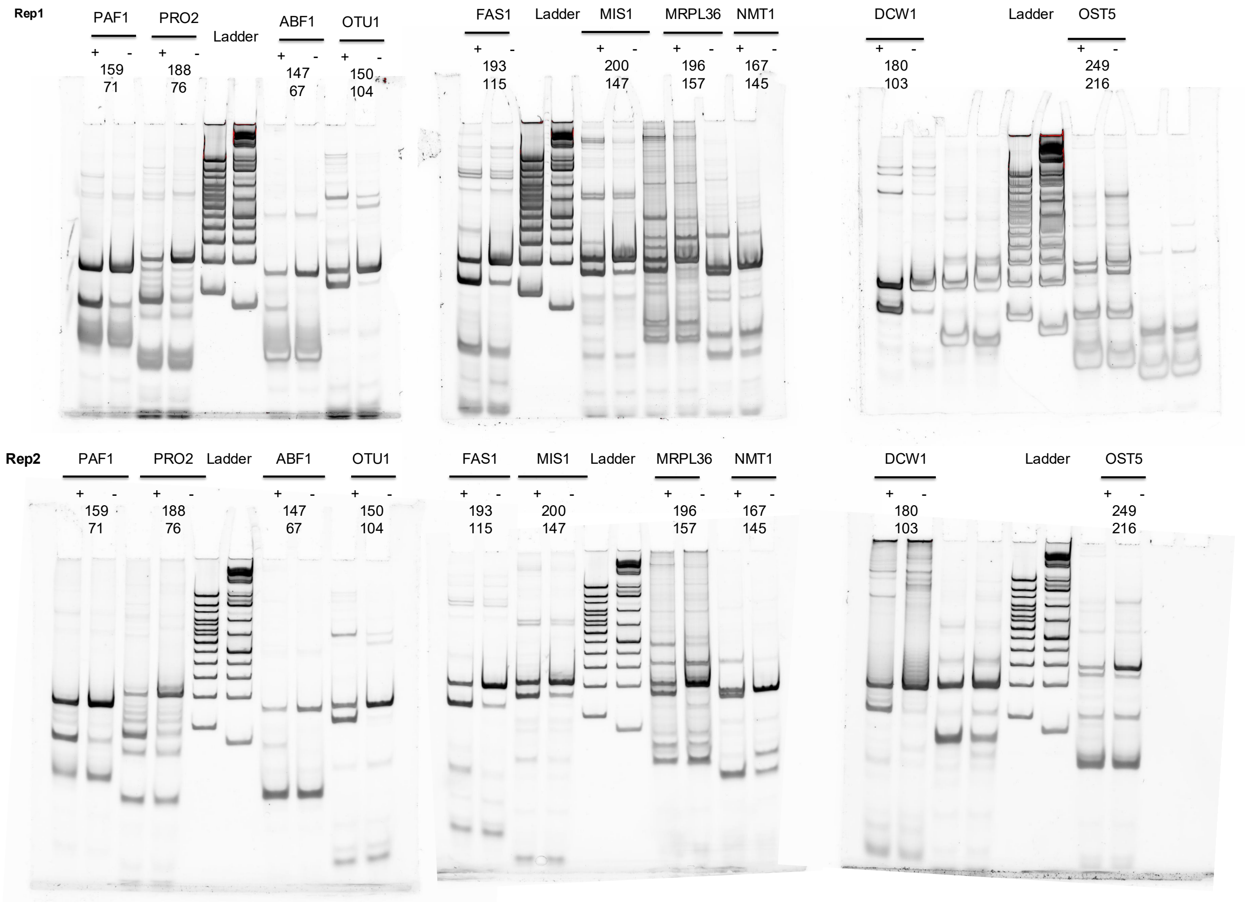

Supplementary Fig.8 | CLAP gels.

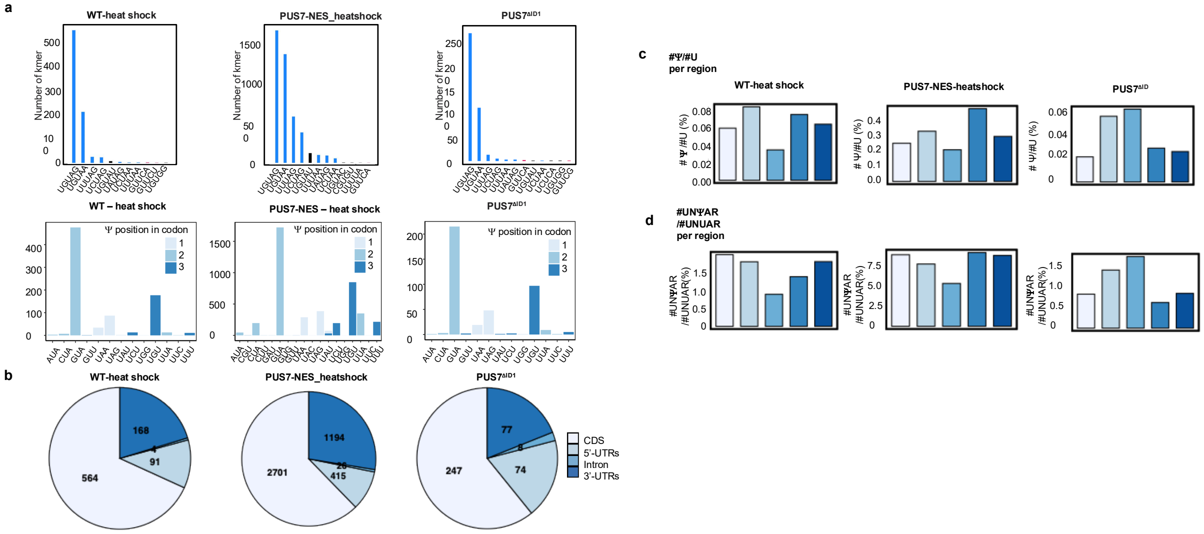

Supplementary Fig.9 | High-confidence Ψ sites for different cells under various stress conditions. a, 5-mer accumulation and codon enrichment for mRNAs isolated from different localized PUS7 cells with U to C mismatch over 40%. **b,** The number of Ψ sites distribution in coding-region, 5'-UTRs, intron, and 3'-UTR for PUS7-control-heat shock, PUS7-NES-heat shock, PUS7$^{\Delta ID}$, respectively. **c,d,** ratio of #Ψ/#U and #UNΨAR/#UNUAR in different regions for PUS7-control-heat shock, PUS7-NES-heat shock, PUS7$^{\Delta ID}$, respectively. (*n* = 2).

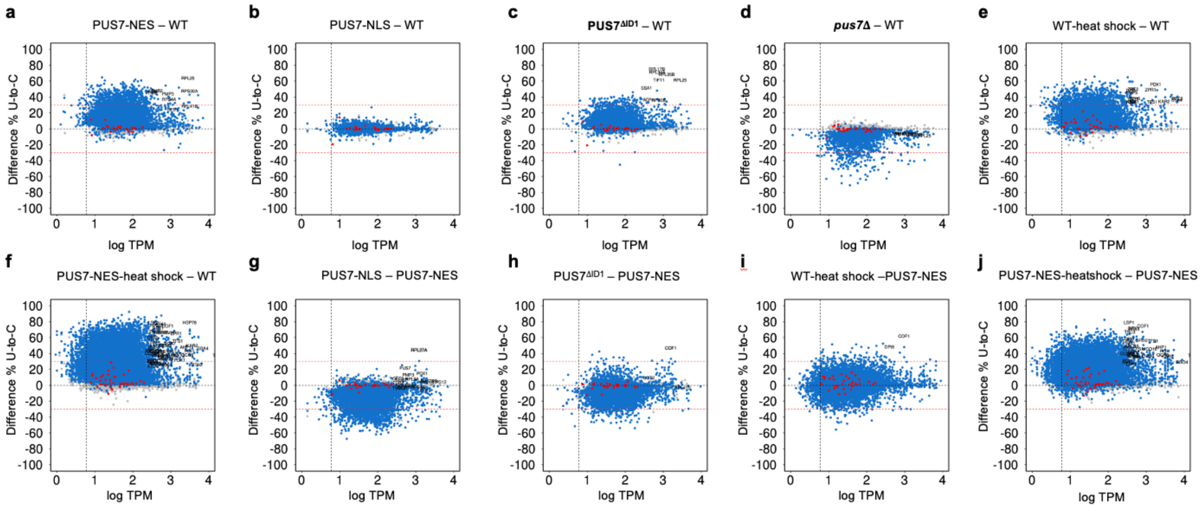

Supplementary Fig. 10 | Potential Ψ-positions determined are plotted according to the difference of U-to-C base-calling error (%) between different PUS7 localized samples and PUS7-control against the number of total direct reads (log$_{10}$TPM). (*n=2*). Sites are compared when over 10 reads coverage is achieved on both groups, and a p-value <0.001 is achieved for at least one group (IVT reads>7 and mm_IVT% < 10%).

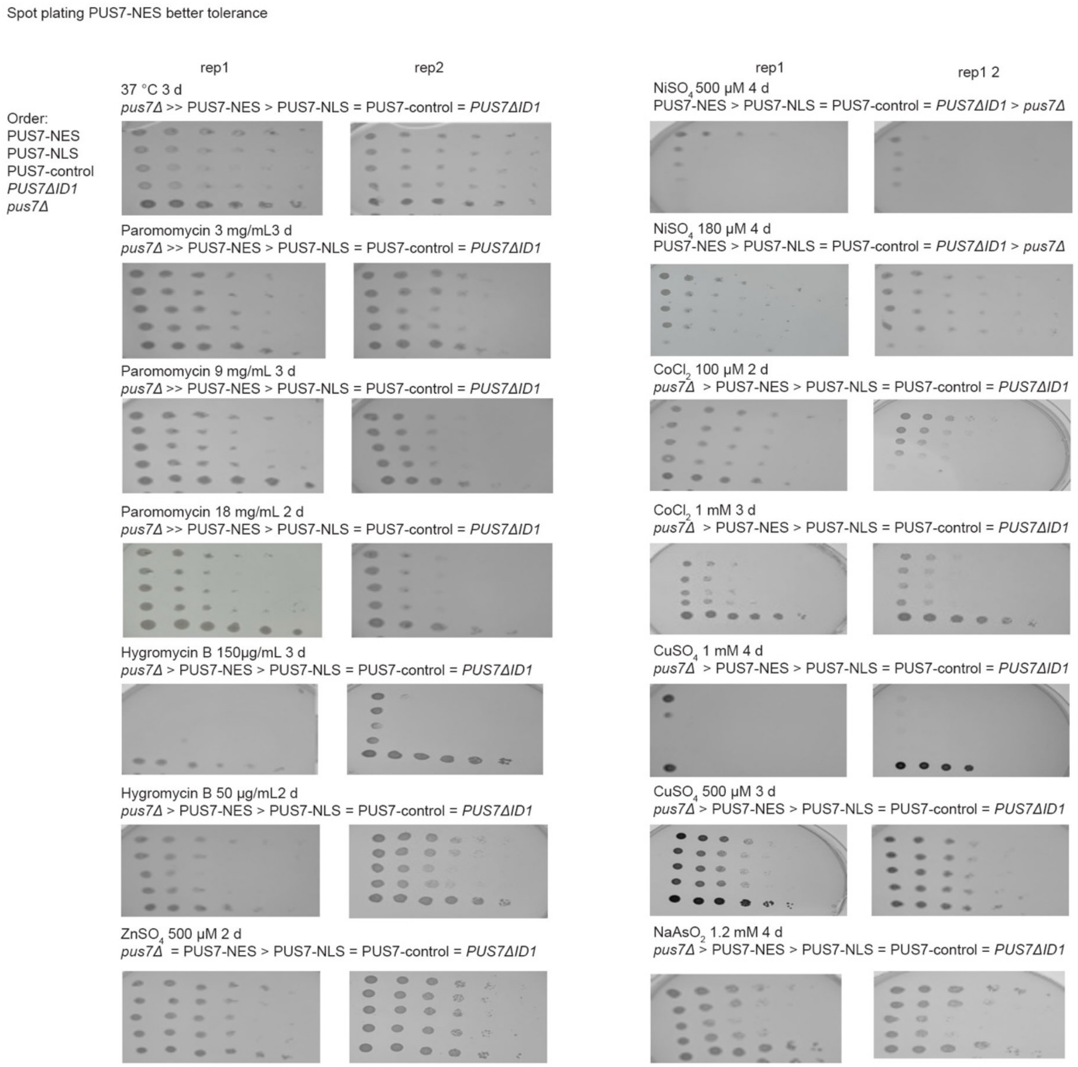

Supplementary Fig.11 | Spot plating assay for PUS7-NES, PUS7-NLS, PUS7-control, PUS7$^{\Delta ID1}$, and *Pus7*Δ yeast with PUS7-NES showing better tolerance. The cells are placed in the plate in the order of PUS7-NES, PUS7-NLS, PUS7-control, PUS7$^{\Delta ID1}$, *Pus7*Δ cells (*n=2*).

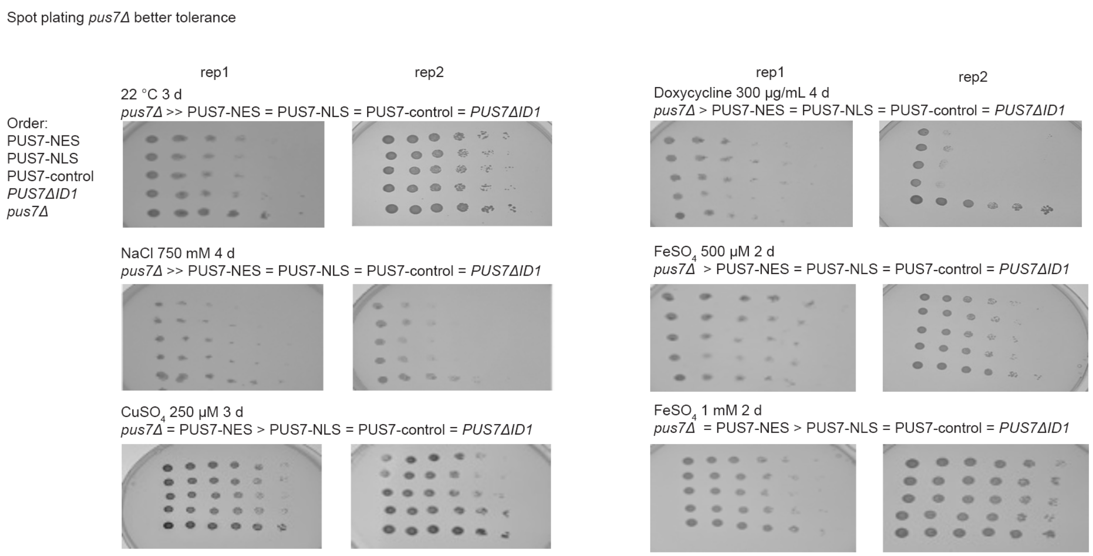

Supplementary Fig.12 | Spot plating assay for PUS7-NES, PUS7-NLS, PUS7-control, PUS7$^{\Delta ID1}$, and *Pus7*Δyeast with *Pus7*Δand PUS7-NES or only *Pus7*Δ showing better tolerance. The cells are placed in the plate in the order of PUS7-NES, PUS7-NLS, PUS7-control, PUS7$^{\Delta ID1}$*,* *Pus7*Δ cells (*n=2*).

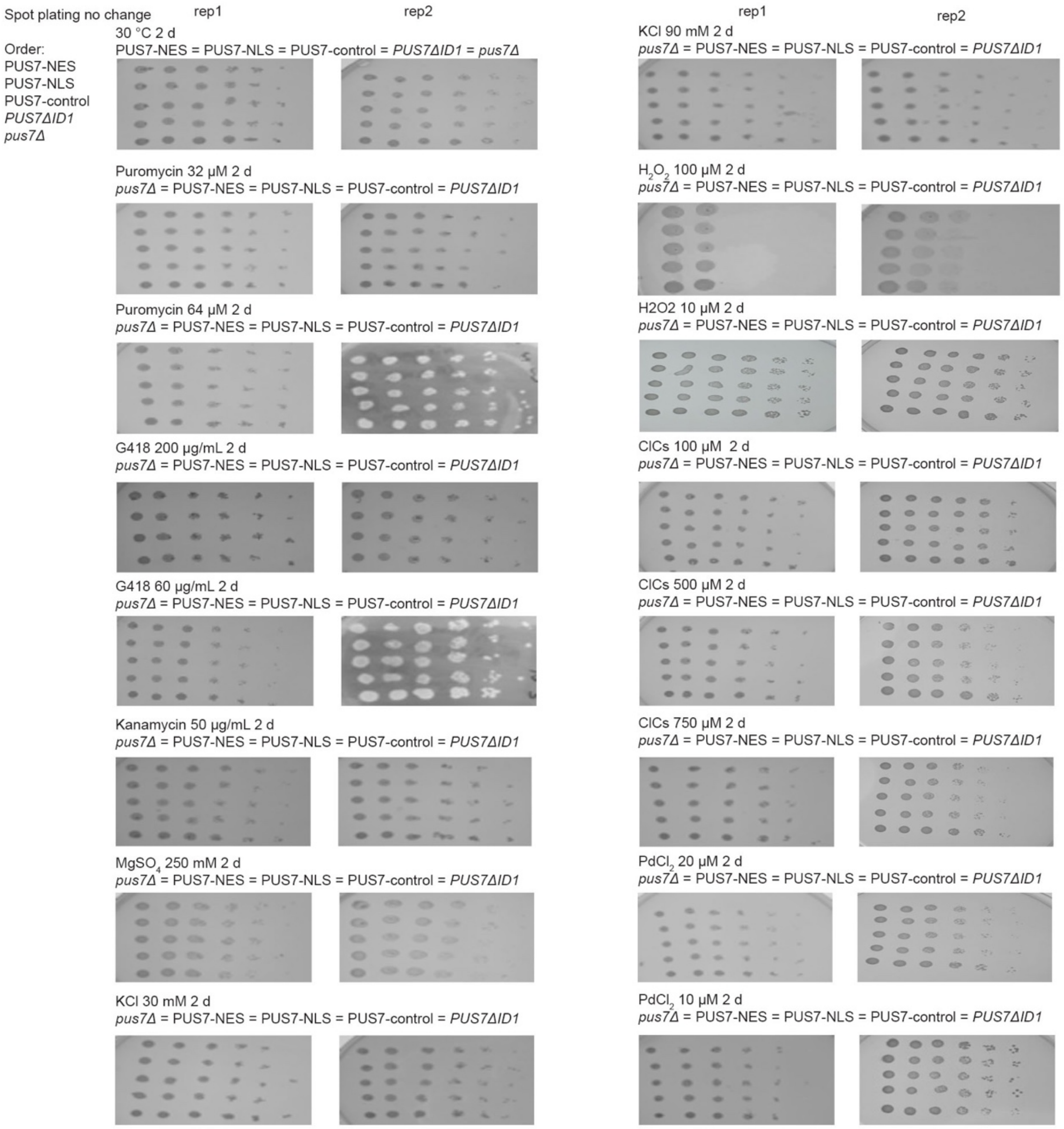

Supplementary Fig.13 | Spot plating assay for PUS7-NES, PUS7-NLS, PUS7-control, PUS7$^{\Delta ID1,}$ and *Pus7*Δ yeast with no difference. The cells are placed in the plate in the order of PUS7-NES, PUS7-NLS, PUS7-control, PUS7$^{\Delta ID1}$*, Pus7*Δ cells (*n=2*).

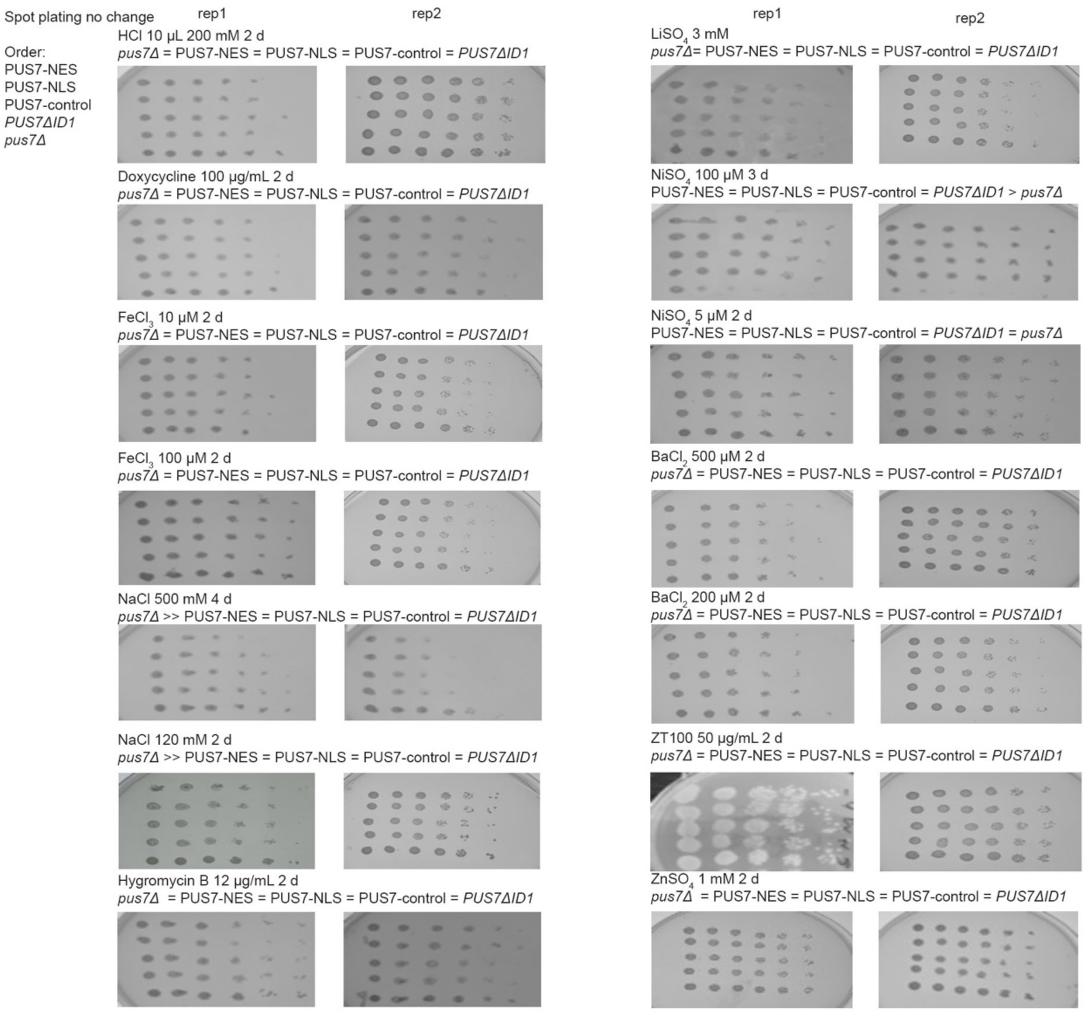

Supplementary Fig.14 | Spot plating assay for PUS7-NES, PUS7-NLS, PUS7-control, PUS7$^{\Delta ID1}$ and *Pus7*Δ yeast with no difference (part2). The cells are placed in the plate in the order of PUS7-NES, PUS7-NLS, PUS7-control, PUS7$^{\Delta ID1}$*,* *Pus7*Δ cells (*n=2*).

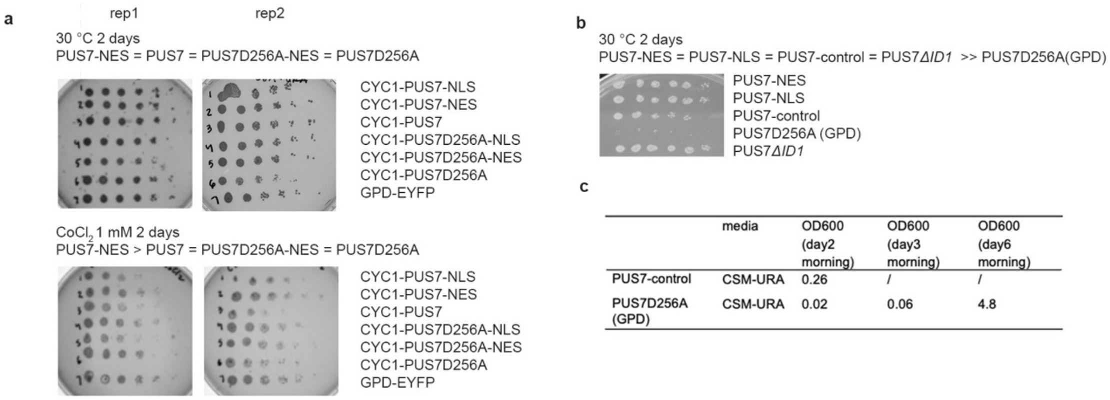

Supplementary Data Fig. 15 | Expression of catalytically inactive PUS7 (D256A) under a low-expression promoter CYC1 in *pus7*Δ BY4741 cells demonstrates that the stress tolerance conferred by PUS7-NES is not due to a scaffold effect. a, Strains expressing PUS7 or catalytically inactive PUS7 under the lower-expression CYC1 promoter show that the stress tolerance conferred by PUS7-NES is not due to its cytoplasmic localization, as PUS7^D256A-NES does not provide a growth advantage. **b-c,** *pus7*Δ BY4741 cells expressing a catalytically inactive version of PUS7 (D256A) under the GPD promoter display general toxicity, as shown by spot plating (**b**) and liquid media growth (**c**), which leads us to switch to the CYC1 promoter to exclude the scaffold effect.

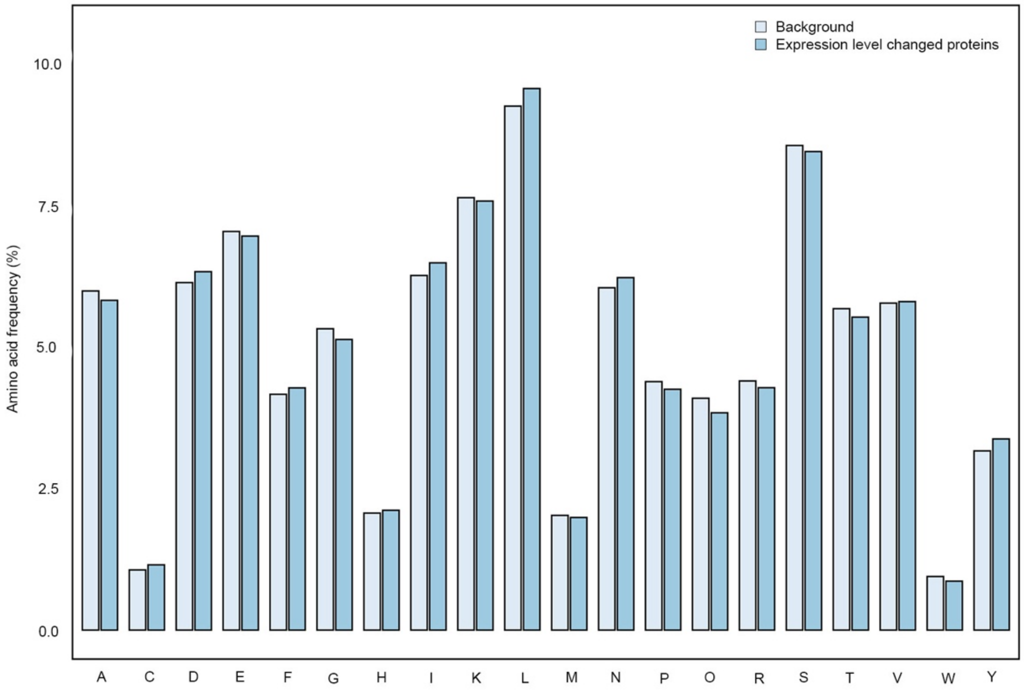

Supplementary Fig. 16 | Distribution of codons within proteins exhibiting altered expression levels between PUS7-NES-Co(II) *vs* PUS7-control-Co(II) *vs* all detected background proteins.

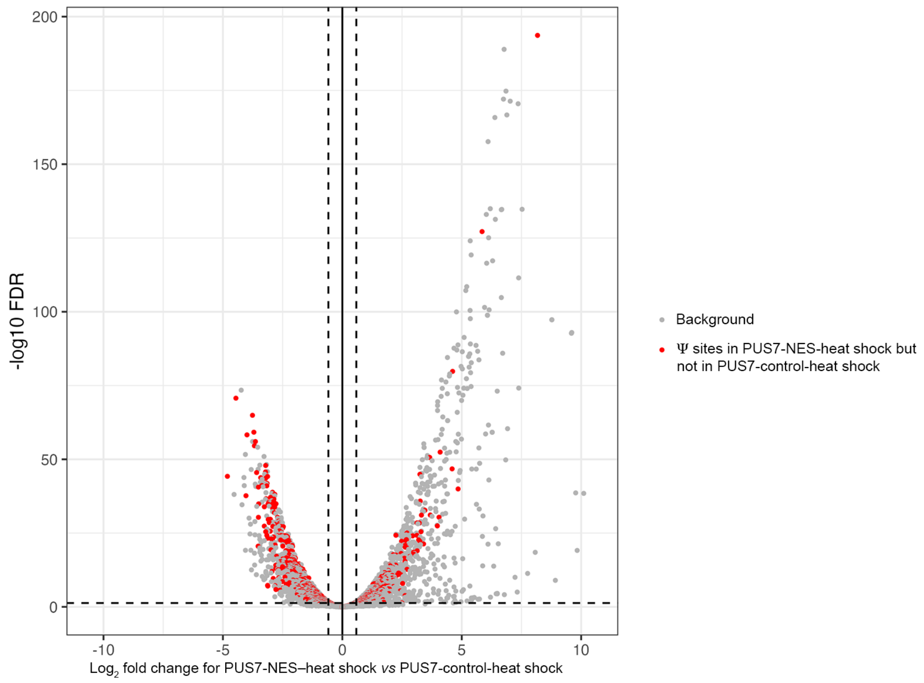

Supplementary Fig. 17 | Volcano plot for PUS7-NES under heat shock *vs* PUS7-NES under normal conditions, with transcripts containing uniquely incorporated Ψ sites in PUS7-NES-heat shock highlighted in red.

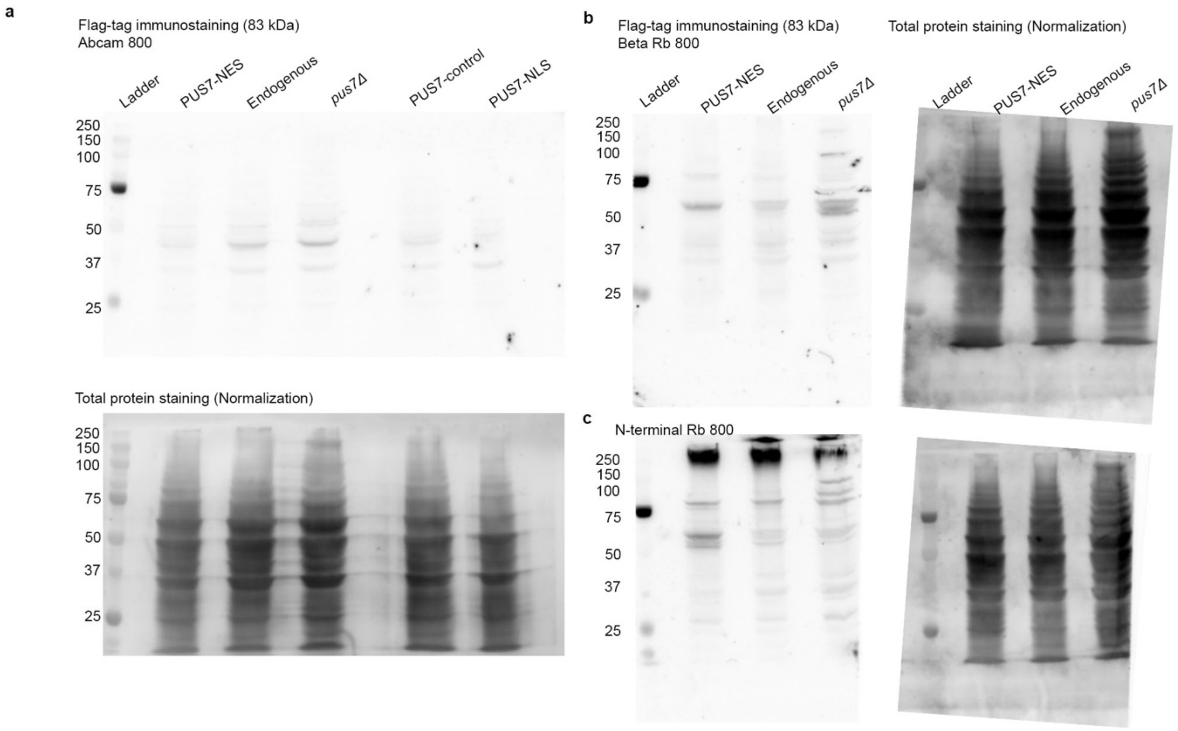

Supplementary Fig. 18 | Negative result of western blot of PUS7 antibodies in yeast. a,

ab224119, Abcam rabbit; **b**, #A305-147A, Bethyl Laboratories; **c**, ABIN310801, antibodies online.

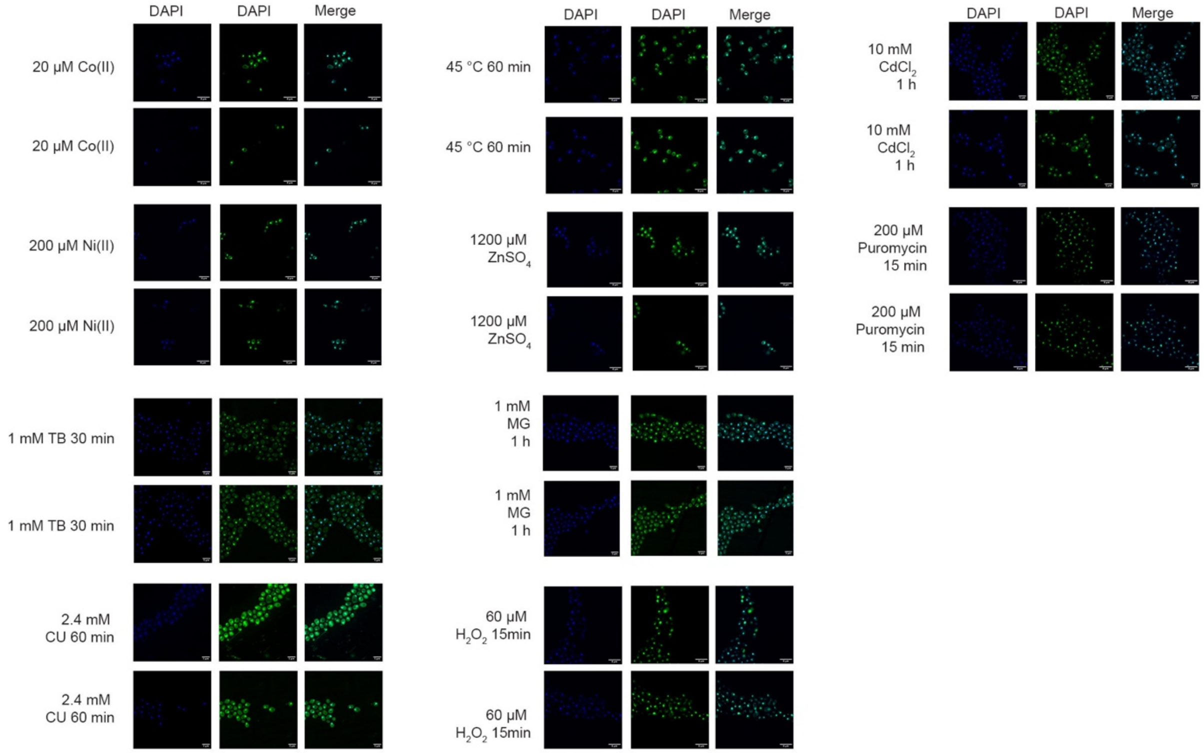

Supplementary Fig. 19 | Additional images of PUS7 localization (PUS7–EYFP) under various stress conditions. Stress conditions include: cobalt (30 µM, 4 h), nickel (200 µM, 4 h), TB (1 mM, 30 min), CU (2.4 mM, 1 h), heat shock (1 h), $ZnSO_4$ (1200 µM, 1 h), MG (1 mM, 1 h), H_2O_2 (60 µM, 15 min), $CdCl_2$ (10 mM, 1 h) and puromycin (200 µM, 15 min). Scale bar: 3 µm.

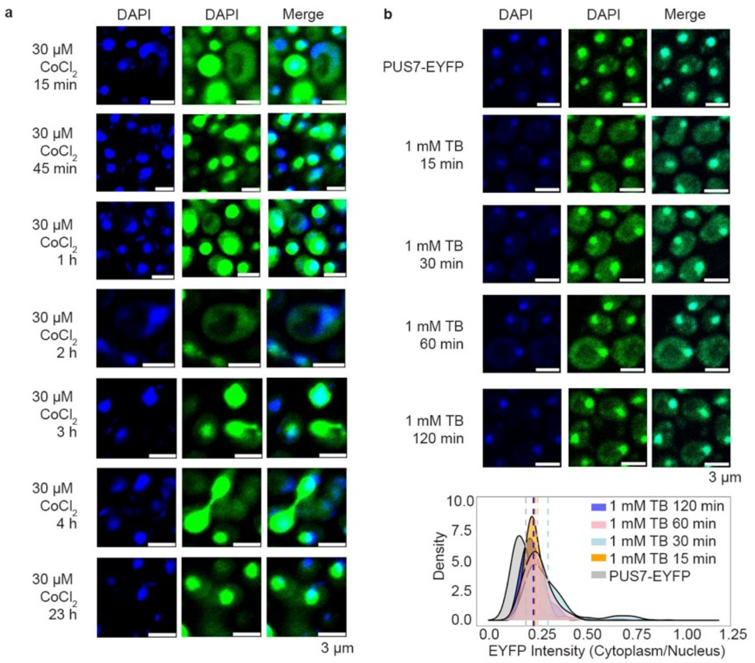

Supplementary Fig. 20 | PUS7 localization changes under stress are time dependent. Visualization and quantification of PUS7 localization (PUS7–EYFP) under 30 µM $CoCl_2$ (**a**), 1 mM TB (**b**), and 2.4 mM CU (**c**) across different time points. Scale bar: 3 µm.

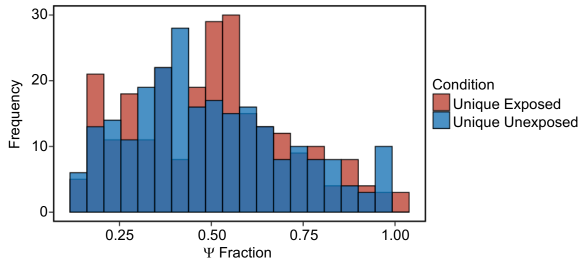

Supplementary Fig. 21 | Distributions of Ψ site fractions that are unique to PM-exposed and unexposed conditions. Histogram showing the frequency of unique Ψ sites for each condition across increasing modification fraction level.

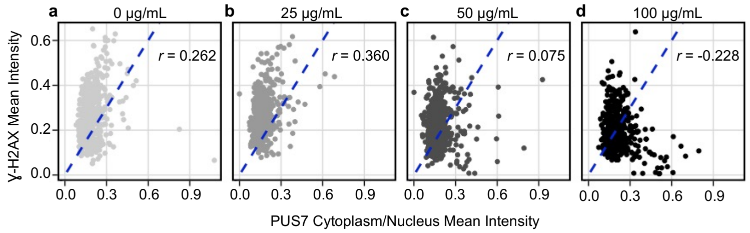

Supplementary Fig. 22 | PUS7 relocalization in BEAS-2B cells exposed to particulate matter is not correlated with DNA damage. Quantification of γ-H2AX mean intensity for individual cells exposed to increasing levels of particulate matter for 24 h, measured through immunofluorescence staining. (*n*=400+ *cells per condition*). Correlation plots showing the relationship between γ-H2AX mean intensity and the cytoplasmic-to-nuclear ratio of PUS7

mean intensity for these individual cells. Dashed blue lines represent y = x. r = Pearson's correlation coefficient.

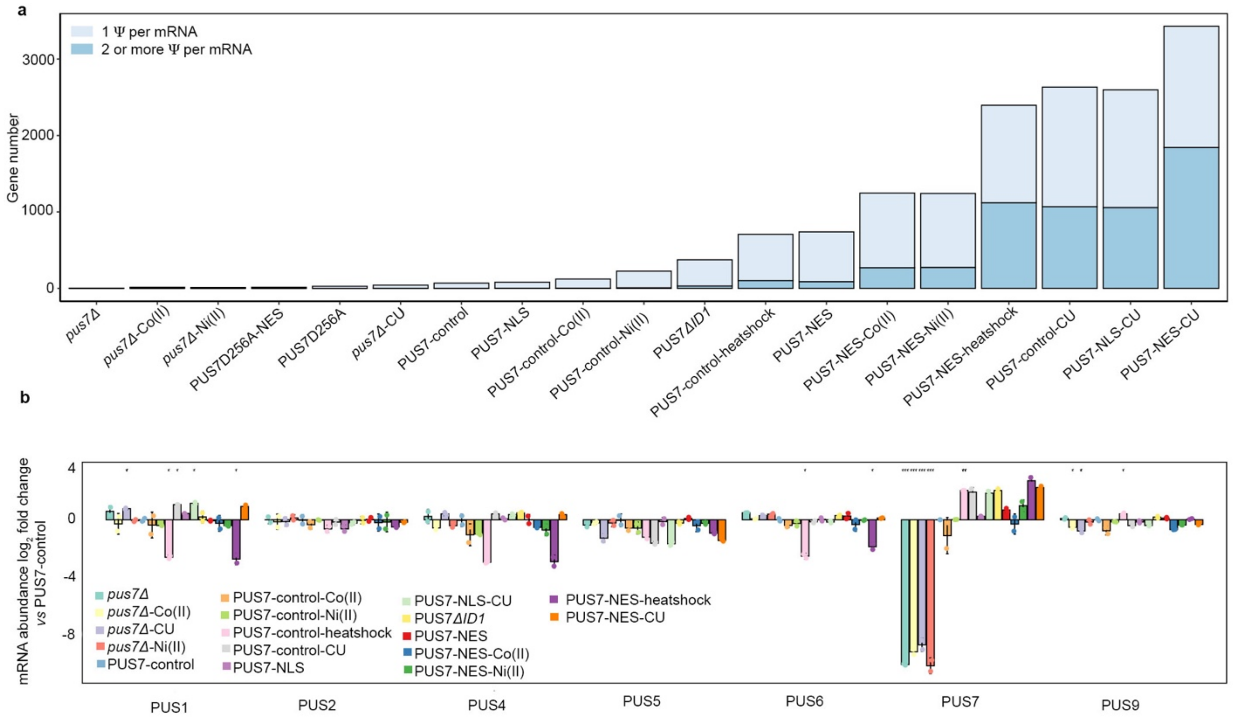

Supplementary Fig. 23 | Ψ distribution and PUS enzyme abundance (TPM) changes under different PUS7 localization and stress conditions. a, Number of genes with one Ψ site versus multiple Ψ sites in different cell groups. **b**, Changes in mRNA abundance for various PUS enzymes when PUS7 localization differs or under heat shock. Data are shown as mean ± SD. Welch's two-sample t-test (unpaired, two-sided) was used. *$P < 0.05$, **$P < 0.01$, ***$P < 0.001$ ($n = 2$).

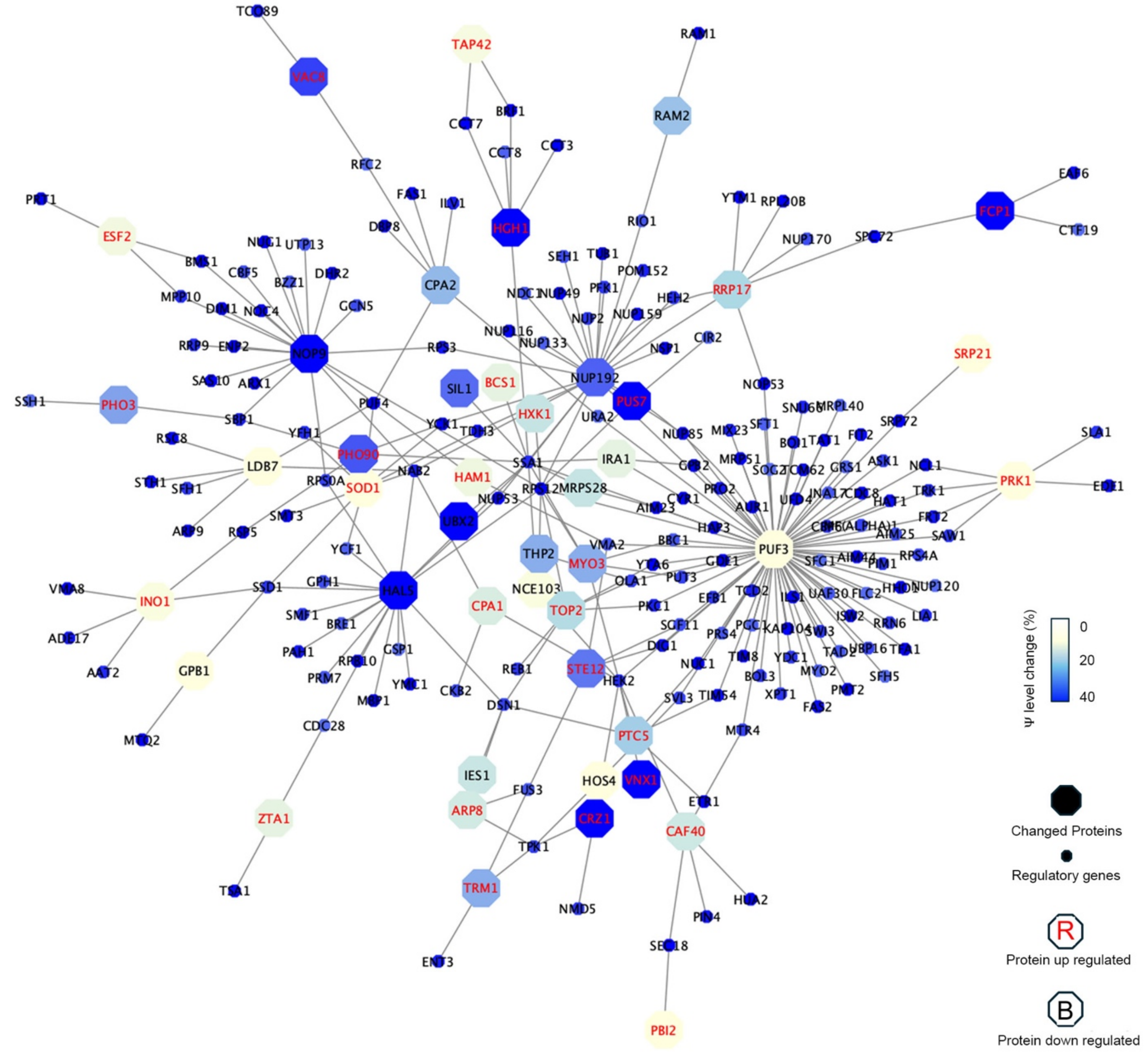

Supplementary Fig. 24 | Physical interactions between expression level changed proteins and proteins whose encoding RNAs are robustly being incorporated by Ψ in PUS7-NES-Co(II) but not PUS7-control-Co(II) (30 µM cobalt stress for 4 h). 48 out of 68 proteins with expression level changes between PUS7-NES-Co(II) and PUS7-control-Co(II) have physical interaction with genes whose encoding RNAs are robustly incorporated by Ψ in PUS7-NES-Co(II) but not PUS7-control-Co(II). Circle color indicates Ψ increasing level (big circle: expression level changed proteins; small circle: physically interacting genes). Upregulated proteins are labeled in red, downregulated proteins are labeled in black.
